# Investigation of neutrophil-to-lymphocyte ratio and mean platelet volume in sudden hearing loss^☆^^☆☆^

**DOI:** 10.1016/j.bjorl.2015.08.009

**Published:** 2015-09-07

**Authors:** Rauf Oğuzhan Kum, Muge Ozcan, Deniz Baklaci, Nurcan Yurtsever Kum, Yavuz Fuat Yilmaz, Adnan Unal, Yonca Avci

**Affiliations:** a Ankara Numune Education and Research Hospital ENT Clinic, Ankara, Turkey; b Department of Otorhinolaryngology, Hitit University, Çorum, Turkey

**Keywords:** Hearing loss, sensorineural, Hearing loss, sudden, Neutrophils, Platelet count, Biological markers, Lymphocyte count, Perda auditiva neurossensorial, Perda auditiva súbita, Neutrófilos, Plaquetas, Marcadores biológicos, Linfócitos

## Abstract

**Introduction:**

Several theories attempt to explain the pathophysiology of sudden hearing loss.

**Objective:**

The objective of this study was to investigate the possible role of inflammation and atherothrombosis in sudden hearing loss patients through the neutrophil-to-lymphocyte ratio and mean platelet volume.

**Methods:**

Study design – retrospective cross-sectional historical cohort. This study was conducted on two groups: one with 59 individuals diagnosed with sudden hearing loss, and other with 59 healthy individuals with the same characteristics of gender and age distribution, neutrophil-to-lymphocyte ratio and mean platelet volume levels were measured in patients diagnosed with sudden hearing loss as well as in the control group, and it was verified whether these results interfered for a better or worse prognosis with treatment of sudden deafness.

**Results:**

Neutrophil-to-lymphocyte ratio levels are much higher in patients diagnosed with sudden hearing loss compared to the control group. Similarly, mean levels of neutrophil-to-lymphocyte ratio are higher in non-recovered versus recovered patients (*p* = 0.001). However, we could not find a correlation with mean platelet volume levels (*p* > 0.05).

**Conclusion:**

Neutrophil-to-lymphocyte ratio is a quick and reliable indicator regarding diagnosis and prognosis of sudden hearing loss; on the other hand, mean platelet volume may be considered a less important indicator in this aspect.

## Resumo

**Introdução:**

Várias teorias tentam explicar a fisiopatologia da surdez súbita (SS).

**Objetivo:**

: O objetivo deste estudo foi investigar o possível papel da inflamação e da aterotrombose nos pacientes de SS através da relação neutrófilos/linfócitos (RNL) e volume plaquetário médio (VPM).

**Método:**

Forma de estudo – coorte histórica com corte transversal (retrospectivo). Este estudo foi realizado com 59 indivíduos portadores de SS e 59 saudáveis, com as mesmas características em distribuição de gênero e idade. Os níveis de VPM e RNL foram medidos nos pacientes diagnosticados com SS e no grupo controle, verificando-se se tais resultados implicavam em um melhor ou pior prognóstico com o tratamento da surdez súbita.

**Resultados:**

Os níveis da RNL são muito mais altos em pacientes com SS, em comparação com o grupo controle. De forma semelhante, níveis médios da RNL são mais altos nos pacientes não recuperados, em comparação com os recuperados (p = 0,001). Essas diferenças entretanto, não foram observadas em relação aos níveis de VPM (p > 0,05).

**Conclusão:**

RNL é um indicador rápido e confiável no que diz respeito ao diagnóstico e prognóstico de SS; por outro lado, VPM pode ser um indicador menos importante neste aspecto.

## Introduction

Sudden hearing loss (SHL) is a disease characterized by a loss of hearing greater than 30 dB in three contiguous frequencies that occurs in less than three days.^1^ The incidence of SHL ranges from 5 to 20 cases per 100,000 individuals.^2^, ^3^ Since the physiopathology of SHL is still unclear, there are many theories as to the origin of this disease, including bacterial, viral and protozoal infections, blood disorders, vascular occlusion, immune disorders, ototoxic drugs, and metabolic conditions.^3^, ^4^, ^5^, ^6^, ^7^

The cochlea is very susceptible to any alteration of bloodstream. Vascular diseases and platelet alterations may cause some cochlear injuries and be related to SHL.^8^

Platelets secrete and express a large number of substances that are crucial mediators of coagulation, inflammation, thrombosis, and atherosclerosis.^9^ The size and functional activity of circulating platelets vary. Larger platelets are often younger, more reactive, and produce more thrombogenic factors. Mean platelet volume (MPV), which is an indicator of platelet activation, is also used as a marker of atherothrombosis,^10^ and may be an important prophylactic and diagnostic tool in thrombotic and prothrombotic cases.

White blood cell (WBC) count is a useful inflammatory biomarker in clinical practice. Even if the WBC is within a normal range, subtypes of WBC, such as neutrophil-to-lymphocyte ratio (NLR), may predict cardiovascular mortality.^11^, ^12^ NLR is an easily measurable laboratory marker that is used to evaluate systemic inflammation and it has also superiority compared to other WBC subtype counts (e.g., neutrophil, lymphocyte, and total leukocyte counts). This superiority may be due to the stability of NLR compared to the other WBC subtype counts, which could be affected by various pathological and physiological conditions. As these factors can alter the individual WBC subtype counts, NLR may remain more stable. Moreover, NLR may represent both inflammatory and immune pathways that exist together in the patients. NLR has been defined as a novel and potential marker to determine inflammation in cardiac and non-cardiac disorders.^11^, ^13^, ^14^, ^15^, ^16^, ^17^, ^18^

Recently, the relationship between MPV and NLR with SHL were investigated individually.^15^, ^19^, ^20^, ^21^ However, to the authors’ knowledge, this is the first study evaluating MPV and NLR together, and in comparison with each other, in the diagnosis and prognosis of the SHL patients. Since NLR is an indicator of inflammation and MPV indicates atherothrombosis, this study aimed to contribute to the literature by investigating the role of inflammation and atherothrombosis in SHL patients by using NLR and MPV, respectively.

## Methods

This cross-sectional historical cohort study included an SHL patient group and a healthy control group. The SHL group included 59 patients who were admitted to a tertiary referral hospital ENT Clinic and were diagnosed with SHL between May of 2010 and December of 2013. Patients with history or clinical findings of any inflammatory, autoimmune, acute or chronic infectious diseases, hypertension, conductive hearing loss, angina pectoris, myocardial infarction, diabetes mellitus, metabolic syndrome, chronic obstructive pulmonary disease, amyloidosis, chronic renal insufficiency obstructive sleep apnea, current smoking, or active otologic disease were excluded, as were those who did not have a type A audiometric tympanogram. The control group included 59 age- and sex-matched healthy individuals who came to the ENT polyclinic for a required examination due to an employment application, had normal audiological findings and no active symptoms.

NLR was calculated as a simple ratio between the absolute neutrophil and the absolute lymphocyte counts. The Beckman Coulter LH 750 automated blood cell counter (Beckman Coulter analyzer, CA, United States) was used for complete blood count (CBC) measurements (Neutrophil, lymphocyte, platelet, NLR, MPV and WBC), which based on technique of laser flow cytometry scattergrams, and were performed within 2 h after blood sampling. Blood samples from the patient group were obtained before the patient was administered any treatment. All samples were run in duplicate, and the mean values were used for statistical analysis.

Audiometric examinations were performed in quiet rooms with an Interacoustics AC-40 clinical audiometer according to the manufacturer instructions, and all the tests were conducted by the same audiometrist. Audiologic data were reported in accordance with the recommended methods of the Hearing Committee of the American Academy of Otolaryngology, Head, and Neck Surgery, which endorsed a new minimal standard for reporting hearing results in clinical trials.^22^ Audiometric examinations were performed initially and after one month of the treatment. SHL patients had a minimum of 30 dB hearing loss at three consecutive frequencies that developed within 72 h and was not associated with other known pathologies, including Meniere's disease, autoimmune disease, ototoxicity, or neoplasm. The hearing loss was unilateral in all patients.

All of the patients were treated with the standard SHL protocol, which included prednisone in the dose of 1 mg/kg per day, with a progressive dose reduction, maintained for at least two weeks. The mean interval of the blood tests following the onset of SHL was 1.98 ± 1.26 days (range 1–6 days)

Patients were classified as follows (according to the recovery observed in one month of follow-up): 1 – Complete: pure tone average (PTA) within 10 dB hearing levels (HL) of initial HL or within 10 dB HL of the HL of the unaffected ear; 2 – Partial: PTA dB HL within 50% of initial HL or greater than 10 dB HL improvement of the HL; and 3 – no recovery: less than 10 dB HL improvement in HL relative to the initial HL 1. Then, the patients were divided into two groups as recovered (Complete + Partial) and unrecovered (No recovery). The patients were also divided into three subgroups according to severity of hearing loss as mild (<40 dB loss for any frequency), moderate (up to 80 dB), and severe (>80 dB).

Age, gender, MPV, NLR, and other laboratory data of the patients were recorded for all groups. The correlations between the MPV and NLR were evaluated in the SHL group and in the control group, and SHL patients were compared according to the recovery and severity of hearing loss. Also, correlation between the MPV and NLR was determined in the SHL patient group.

The study was approved by the local ethics committee and conducted in accordance with the ethical principles described by the Declaration of Helsinki. An informed consent was obtained from all participants prior to their participation in the study.

SPSS for Windows, version 15.0, software was used for all data analyses. Data distributions were analyzed with the Shapiro–Wilk test. Student's *t*-test for independent samples was used to analyze age, MPV values, and platelet count. In cases of normal distribution, Mann–Whitney's *U* test was used to compare the groups (gender). The correlations between the variable pairs were analyzed using Pearson's correlation test. A level of *p* < 0.05 was considered significant.

## Results

There was no significant difference between the age or gender in the SHL and control groups. The mean age of patients and controls were 46.10 ± 11.91 and 42.84 ± 11.85 years, respectively. The male-to-female ratio in the SHL group was 38:21, and 31:28 in the control group (Table 1).

**Table 1:** Intergroup comparison of age, gender, mean platelet volume (MPV), neutrophil-to-lymphocyte ratio (NLR) values, and platelet counts of the patients and the control group.

Variables	Patient (*n* = 59) Mean ± SD	Control (*n* = 59) Mean ± SD	*p*-Value
*Age (years)*	46.10 ± 11.91	42.84 ± 11.85	0.140

*Gender*			0.193
Female	21 (35.6%)	28 (47.5%)	
Male	38 (64.4%)	31 (52.5%)	

*NLR*	3.24 ± 2.26	1.53 ± 0.47	0.001^a^
*MPV (fL)*	9.83 ± 1.50	9.98 ± 0.07	0.470
*Platelet (103* *U)*	249.44 ± 48.16	244.86 ± 47.25	0.603
*WBC (103* *U)*	8.39 ± 2.83	6.29 ± 0.973	0.001^a^
*Neutrophil (103* *U)*	5.64 ± 2.55	3.43 ± 0.71	0.001^a^
*Lymphocyte (103* *U)*	2.06 ± 0.77	2.35 ± 0.53	0.029^a^

SD, standard deviation; WBC, white blood cell.

a *p* < 0.05.

The laboratory data of patients and controls are shown in Table 1. The mean MPV value was 9.83 ± 1.50 fL in the patients and 9.98 ± 0.07 fL in the control group, and the difference was not statistically significant (*p* = 0.470). The mean NLR value was 3.24 ± 2.26 in the SHL patients and 1.53 ± 0.47 in the control group, and the difference was statistically significant (*p* = 0.001; Table 1).

The laboratory data of the SHL group according to response to the treatment as recovered and unrecovered groups are shown in Table 2. The mean MPV value was 9.91 ± 1.49 fL in the unrecovered group and 9.75 ± 1.52 fL in the recovered group, and the difference was not statistically significant (*p* = 0.785). The mean NLR value was 4.45 ± 2.63 in the unrecovered group and 2.15 ± 1.04 in the recovered group, and the difference was statistically significant (*p* = 0.001; Table 2).

**Table 2:** Comparison of laboratory data between the recovered and unrecovered patients.

Variables	Unrecovered (*n* = 31) Mean ± SD	Recovered (*n* = 28) Mean ± SD	*p*-Value
NLR	4.45 ± 2.63	2.15 ± 1.04	0.001^a^
MPV (fL)	9.91 ± 1.49	9.75 ± 1.52	0.785
Platelet (103 U)	248.50 ± 48.69	250.86 ± 48.47	0.767
WBC (103 U)	9.20 ± 3.27	7.67 ± 2.16	0.118
Neutrophil (103 U)	6.76 ± 2.92	4.64 ± 1.65	0.006^a^
Lymphocyte (103 U)	1.80 ± 0.77	2.30 ± 0.70	0.012^a^

SD, standard deviation; MPV, mean platelet volume; NLR, neutrophil-to-lymphocyte ratio; WBC, white blood cell.

a *p* < 0.05.

The mean NLR and MPV values according to severity of hearing loss are shown in Table 3. A statistically significant correlation was observed between NLR values and severity of hearing loss (*p* = 0.015). The mean NLR values in the patients with severe SHL were significantly higher than in the mild SHL group (*p* = 0.004). However, there was no correlation between MPV values and severity of hearing loss (*p* = 0.701).

**Table 3:** Mean analysis for NLR and MPV according to severity of hearing loss as mild (<40 dB), moderate (up to 80 dB), and severe (profound, >80 dB).

	1 Mild (*n* = 24)	2 Moderate (*n* = 24)	3 Severe (*n* = 11)	*p*
	Mean ± SD	Mean ± SD	Mean ± SD	
MPV (fL)	9.83 ± 1.58	9.74 ± 1.56	10.00 ± 1.27	0.701
NLR	2.35 ± 1.14	3.25 ± 2.06	5.15 ± 3.33	0.015^a^
				1–2, *p* = 0.117
				2–3, *p* = 0.092
				1–3, *p* = 0.004^a^

SD, standard deviation; MPV, mean platelet volume; NLR, neutrophil-to-lymphocyte ratio.

a *p* < 0.05.

No correlation was observed between MPV and NLR in the SHL group (*r* = 0.190; *p* = 0.149).

## Discussion

The key to successful treatment of diseases lies in the understanding of their etiopathogenesis. Since the physiopathology of SHL is still unclear, there are conflicting theories regarding its true cause.^8^, ^23^, ^24^ There are many theories as to the origin of this disease, including viral infections, inflammation, blood disorders, vascular causes, immune disorders, and rupture of the labyrinthine membrane.^12^, ^25^, ^26^ Since the cochlea is mainly supplied by a single, terminal artery (the labyrinthine artery), the inner ear is very much prone to circulatory alterations.^27^ In vascular cases, the problems may be present on the blood vessel wall, as is the case in arteritis and spasms, or they may be intravascular. A history of vascular disease or platelet alterations is the only clinical finding that could lead one to infer a vascular cause of SHL. Although the most widely accused factor in the etiology is viral inflammation of the neural fibers and ganglia,^28^ this has not been proven yet.

NLR is an easily measured biomarker that correlates with clinical status. It is calculated from CBC, and is an inexpensive, easy to obtain, widely available marker of inflammation that can aid in the risk stratification of patients with various diseases.^1^, ^13^, ^29^, ^30^ It is as valuable as some high-cost inflammatory markers, including IL-6, IL-1β, IL-8, and TNF-α.^31^ Conversely, MPV can be used as marker for high platelet activity, which takes an active role in the pathophysiology of thrombosis, coagulation, and atherosclerosis.^32^

In recent studies, NLR was determined to be a reliable marker for the diagnosis and prediction of the SHL and Bell's palsy prognoses.^14^, ^15^, ^33^ Ulu et al. reported that when the patients with SHL were compared according to the recovery, NLR levels were higher in patients who did not recover. This may be explained with the higher inflammatory situation in unrecovered patients, and this result may help clinicians caring for SHL patients with higher NLR levels in terms of treatment and prognosis.^15^ In the previous study by Masuda et al., neutrophil and levels of inflammatory markers were higher in SHL patients.^34^ Most recently, Ozler classified SHL patients according to severity of hearing loss as mild (<40 dB loss for any frequency), moderate (up to 80 dB), and severe (>80 dB), and found that the mean NLR values in patients with SHL were significantly higher than in the control group, and that the mean NLR values in patients with severe sensorineural hearing loss were significantly higher than in the other groups.^35^ In the present study, NLR values were significantly higher in patients with SHL when compared with the control group. Similarly, the mean NLR was higher in the unrecovered patients when compared with those who recovered; a significant correlation was observed between NLR values and severity of hearing loss, indicating the presence of inflammation.

Platelets play an active role in the pathophysiology of thrombosis, coagulation, inflammation, and atherosclerosis.^9^, ^36^ Upon activation, platelets release vasoactive and thrombogenic agents, which are important factors in the formation of thrombosis in the vessel. Several investigators have used a series of platelet indices that are measured by hematology analyzers, since platelet activation leads to morphologic changes.^37^ Large platelets are metabolically and enzymatically more active and have a higher thrombotic potential.^38^ Higher MPV values are beneficial markers of higher platelet activity and were found to be associated with atherothrombosis. In addition, MPV is one of the most extensively studied platelet activation markers.^32^, ^39^

MPV values were studied in patients with SHL, yielding controversial results. In the recent studies by Ulu et al. (with 40 patients and controls) and by Sagit et al. (with 31 patients and controls), the mean MPV values in SHL patients were 10.5 ± 0.9 fL and 9.01 ± 1.24 fL, respectively, which were significantly higher than the values of the control groups (9.6 ± 0.5 fL and 8.21 ± 0.76 fL, respectively).^20^, ^21^ In another study by Karli et al. (with 46 patients and controls), the mean MPV value was 8.25 ± 0.86 fL in SHL patients and 7.98 ± 0.87 fL in the control group, which was not significantly different, and they concluded that there was no microvascular response in the etiology of SHL.^19^ In the present study (with 59 patients and controls), the mean MPV value was 9.83 ± 1.50 fL in SHL patients and 9.98 ± 0.07 fL in the control group; similarly, the mean MPV value was 9.91 ± 1.49 fL in the unrecovered group, and 9.75 ± 1.52 fL in the recovered group, and there was no significantly difference between the groups. The absence of any correlation of MPV with any of the studied parameters may suggest that microvascular response is a suspicious theory in etiopathogenesis of SHL.

In a large epidemiologic study with 326 patients by Demirin et al., the mean MPV value in normal Turkish adults was 8.9 ± 1.4 fL, and 95% of the individuals had an MPV between 7.2 and 11.7 fL.^40^ If the MPV values of the control group in this study were equivalent to those of the study by Demirin et al., the present results would have been considered significant.

## Conclusion

The results of the present study indicated that, when the SHL patients were compared according to the recovery, NLR levels were higher in patients who did not recover. However, no such correlation was observed between MPV levels and patient recovery. This may indicate that inflammation plays a larger role in the pathogenesis of SHL than the microvascular response. In the evaluation of SHL, NLR is a quick and reliable marker for predicting the diagnosis and prognosis, while MPV may be a less important marker. Although the present sample size was larger than that used in previous studies, this issue should be investigated in a larger patient cohort. Because this area of research is a novel field, prospective, multicenter, controlled studies are needed.

## Conflicts of interest

The authors declare no conflicts of interest.
